# Breast Cancer Outcomes From Ductal Carcinoma In Situ: A Population‐Based Cohort Study

**DOI:** 10.1002/ijc.70559

**Published:** 2026-05-20

**Authors:** Qian Chen, Ian Campbell, Mark Elwood, Alana Cavadino, Phyu Sin Aye, Sandar Tin Tin

**Affiliations:** ^1^ Department of Epidemiology and Biostatistics, Faculty of Medical and Health Sciences University of Auckland Auckland New Zealand; ^2^ Department of Surgery, Faculty of Medical and Health Sciences University of Auckland Auckland New Zealand; ^3^ Department of Pharmacology, Faculty of Medical and Health Sciences University of Auckland Auckland New Zealand; ^4^ Cancer Epidemiology Unit, Oxford Population Health University of Oxford Oxford UK

**Keywords:** ductal carcinoma in situ, invasive ipsilateral breast cancer, landmark, locoregional treatment, surveillance

## Abstract

This population‐based cohort study investigated whether the subsequent risk of breast cancer after ductal carcinoma in situ (DCIS) has changed over time in New Zealand women diagnosed between 2000 and 2022, using data from the New Zealand Breast Cancer Foundation National Register. The primary outcome was ipsilateral breast event, including invasive cancer (iIBC) or DCIS (iDCIS). Fine‐Gray subdistributional hazard models assessed associations with demographic and clinical factors, and a 5‐year landmark analysis evaluated subsequent risk among women who remained event‐free at 5 years. Among 5830 patients (median follow‐up 4.8 years), the overall 5‐year cumulative risk of iIBC was 3.3% (95% CI: 2.7%, 3.9%). In the 5‐year landmark cohort, the subsequent 5‐year risk was 4.1% (3.3%, 5.0%). Corresponding iDCIS rates were 2.5% (2.0%, 3.0%) and 1.7% (1.2%, 2.3%), respectively. Age < 45 years at diagnosis and DCIS size > 20 mm were associated with a higher iIBC risk, but the association with younger age attenuated in the landmark analysis. Compared with breast conserving surgery (BCS) alone, both radiotherapy following BCS and mastectomy were associated with a lower iIBC risk; however, the association for radiotherapy was not evident at the 5‐year landmark. Overall, women with DCIS had a 5.25‐fold higher risk of invasive breast cancer than the general population (95% CI: 4.79, 5.73). Our findings support the importance of long‐term mammographic surveillance, particularly for women with larger DCIS, those treated with BCS, and possibly younger women.

## What's New?

Women with ductal carcinoma in situ have an elevated risk of subsequent invasive breast cancer, but how this risk evolves over time is unclear. In this population‐based study of ethnically diverse women diagnosed between 2000 and 2022 in New Zealand, the risk of invasive ipsilateral breast cancer persisted beyond 5 years. Younger age and larger ductal carcinoma in situ size were associated with higher risk. Compared with breast‐conserving surgery alone, radiotherapy following breast‐conserving surgery and mastectomy were associated with lower risk, although the association for radiotherapy attenuated beyond 5 years. These findings support prolonged mammographic surveillance, particularly for high‐risk groups.

## Graphical abstract

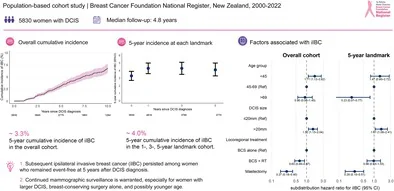

**Abbreviations**
BCFNR: Breast Cancer Foundation National Register
BCS: breast conserving surgery
cDCIS: contralateral ductal carcinoma in situ
cIBC: contralateral invasive breast cancer
DCIS: ductal carcinoma in situ
ER: eestrogen receptor
ET: endocrine therapy
IBE: Ipsilateral breast event
ICD‐10: International Statistical Classification of Diseases, 10th Revision
iDCIS: ipsilateral ductal carcinoma in situ
iIBC: ipsilateral invasive breast cancer
NZCR: New Zealand Cancer Registry
NZDep: New Zealand deprivation
OS: overall survival
RT: radiotherapy
sHR: subdistributional hazard ratio
SIR: standardized incidence ratio

## 1 Introduction

The incidence of ductal carcinoma in situ (DCIS) has increased since the introduction of population‐based mammographic screening, and now accounts for approximately a quarter of screen‐detected breast cancers (invasive and in situ) [1]. The majority of women with DCIS are managed by surgery, with or without radiotherapy (RT) and/or endocrine therapy (ET) to prevent progression to invasive cancer [2, 3, 4]. Despite a generally favorable survival prognosis [5], a UK study found that women with a prior DCIS diagnosis had 2 to 4 times the incidence of invasive breast cancer and breast cancer mortality compared to the general population [6, 7].

Age at diagnosis, DCIS size and the type of locoregional treatment are known risk factors for subsequent ipsilateral breast event (IBE), including invasive (iIBC) or DCIS (iDCIS) [6, 7, 8, 9]. Postoperative RT following breast‐conserving surgery (BCS) has consistently been shown to reduce IBE across different subgroups [10]. However, evidence from EORTC 10853 [11] and SweDCIS [12] trials comparing BCS with or without RT suggests that its effect is the greatest within the first few years post‐surgery. Similarly, the elevated risk of IBE among women diagnosed before age 50 or with DCIS size larger than 25 mm has been found to be more pronounced during this early postoperative period [13]. Several studies have found that the incidence of iDCIS declined after 5 years post‐diagnosis, whereas the risk of iIBC continued to rise over time [12, 13, 14]. Whether similar changes have occurred among ethnically diverse women in New Zealand remains unclear.

New Zealand Māori and Pacific women experience one of the highest breast cancer incidence rates in the world; this disparity is possibly due to higher rates of obesity and lifestyle‐related risk factors [15, 16]. A report from the New Zealand Breast Cancer Foundation National Register (BCFNR) showed that nearly all women diagnosed with DCIS between 2003 and 2020 underwent surgery, with an overall 10‐year locoregional recurrence (invasive and DCIS) free survival of 95% [17]. The lower estimate, compared with international studies, may be explained by sole reliance on registry‐recorded “locoregional recurrence”. In this population‐based study, we considered all subsequent breast cancer events after diagnosis of DCIS and investigated whether the risks changed over time. We also assessed the invasive breast cancer risk in women with DCIS relative to the general population and examined their survival outcomes.

## 2 Methods

### 2.1 Data Sources

This study used data from the BCFNR for the period 2000–2022. The register started prospective breast cancer data collection from Auckland in 2000, and Waikato in 2005 (data retrospectively added back to 1991), expanded to Christchurch in 2009 and to Wellington in 2010, covering approximately 63% of New Zealand's breast cancer cases [17]. Since 2020, the register has expanded to include all breast cancer diagnoses nationwide [18]. BCFNR collects data from multiple sources, including the New Zealand Cancer Registry (NZCR), which primarily records biopsy‐confirmed cancers, and other sources, such as multidisciplinary meeting lists, referral letters, clinical notes, and histopathological reports to identify new diagnoses, local and distant recurrences, as well as to collect other clinicopathological information. Data collected from clinical records are audited where possible against other national data sets such as the National Minimum Dataset and Pharmaceutical Collection annually. Mortality data are verified through the Ministry of Health Mortality Collection. This register uses an opt‐out approach and has a withdrawal rate of less than 1% [17]. We included women with unilateral DCIS (International Classification of Diseases 10th revision (ICD‐10) diagnosis code D051) diagnosed between 2000 and 2022. Patients with a prior diagnosis of either DCIS or invasive breast cancer before their index diagnosis of unilateral DCIS were excluded (Figure S1).

### 2.2 Variables of Interest

Demographics and clinical information were obtained from the BCFNR. Variables of interest included age at diagnosis, ethnicity, year of diagnosis (2000–2010, 2011–2022), socioeconomic status (represented by the New Zealand deprivation (NZDep) index, which was based on the domicile code, with 1 representing the least deprived areas and ten representing the most deprived areas [19]), area of residence (rural, urban/other, according to Statistics NZ definitions based on the domicile code [20]), mode of diagnosis (program screening, non‐program image, symptomatic), laterality (left, right), tumor grade, pathohistological DCIS size (< 20 mm, ≥ 20 mm), tumor necrosis (absent, present, unknown), type of final surgery, circumferential margins, eestrogen receptor (ER) status and adjuvant ET (ER‐positive with ET, ER‐positive without ET, ER‐negative, not tested).

### 2.3 Outcomes

The primary outcome was the subsequent diagnosis of an IBE, including invasive cancer or DCIS. The secondary outcomes included contralateral invasive breast cancer (cIBC), contralateral DCIS (cDCIS), breast cancer mortality, and overall survival (OS). IBE was defined as the first occurrence of DCIS or invasive cancer in the ipsilateral breast. Regional lymph node involvement or distant metastasis without concurrent cancer in the breast was classified as iIBC. Bilateral breast events were included in separate analyses for both IBE and CBC. All breast event endpoints were measured from the date of tissue diagnosis to the date of first breast cancer event, death, last follow‐up, or 31 December 2024. Breast cancer‐specific mortality was defined as the time from diagnosis to death from breast cancer; OS as the time from diagnosis to death from any cause.

### 2.4 Statistical Methods

Differences in patients' demographic and treatment characteristics across subgroups were assessed using chi‐square (χ2) tests for categorical variables and the Kruskal–Wallis test for continuous variables. Cumulative incidence of each outcome was estimated using the cumulative incidence function, with death as a competing risk. To identify the instantaneous risk of IBE and CBC, the hazard rates were estimated using nonparametric smoothing methods [21]. Landmark analyses at 1‐, 3‐, and 5‐year landmarks post‐diagnosis were performed among patients with follow‐up longer than these time points to avoid immortal time bias [22]. Multivariable Fine‐Gray subdistribution models were used to estimate subdistribution hazard ratios (sHR) [23] for factors associated with iIBC. Subgroup analyses estimated the 5‐year cumulative incidence of iIBC by diagnosis period (2000–2010 vs. 2011–2022) overall and in the two regions with data available from 2000 onward (Auckland and Waikato). Standardized incidence ratios (SIRs) were calculated as the ratio of the observed incidence of invasive breast cancer in DCIS survivors to the expected incidence among New Zealand women in general; for the latter, age‐specific incidence rates were obtained from the NZCR [24]. Poisson regression was used to compute 95% confidence intervals (95% CI) for the SIR. The absolute survival rate was estimated using the Kaplan–Meier method. All analyses were performed using R 4.3.1 [25], and a two‐sided *p* value less than 0.05 was considered statistically significant.

## 3 Results

### 3.1 Patient Characteristics

A total of 5830 women diagnosed with unilateral DCIS as their first breast cancer were included in the study (Table 1). Most women were aged 45–69 years (82.7%), identified as European (71.0%), resided in the least deprived area (49.2%), urban/other areas (86.3%) and were diagnosed through program screening (60.4%). Overall, 37.7% of women received BCS with RT, 34.3% underwent mastectomy, 26.5% had BCS alone, and 1.5% did not receive surgery.

**TABLE 1:** Patient characteristics by type of treatment.

Characteristic	Overall *N* = 5830	BCS alone *N* = 1544	BCS with RT *N* = 2196	Mastectomy *N* = 2000	No surgery *N* = 90
Age at diagnosis Median (Min, Max)	56 (23.97)	58 (23.92)	57 (28.83)	54 (27.90)	62 (35.97)
Age group					
< 45	466 (8.0%)	98 (6.3%)	104 (4.7%)	259 (13.0%)	5 (5.6%)
45–69	4820 (82.7%)	1255 (81.3%)	1959 (89.2%)	1552 (77.6%)	54 (60.0%)
> 69	544 (9.3%)	191 (12.4%)	133 (6.1%)	189 (9.5%)	31 (34.4%)
Ethnicity					
Māori	479 (8.2%)	119 (7.7%)	199 (9.1%)	152 (7.6%)	9 (10.0%)
Pacific	278 (4.8%)	77 (5.0%)	94 (4.3%)	90 (4.5%)	17 (18.9%)
Asian	828 (14.2%)	241 (15.6%)	299 (13.6%)	279 (14.0%)	9 (10.0%)
European	4142 (71.0%)	1080 (69.9%)	1568 (71.4%)	1444 (72.2%)	50 (55.6%)
Other or Unknown	103 (1.8%)	27 (1.7%)	36 (1.6%)	35 (1.8%)	5 (5.6%)
Period of diagnosis					
2000–2010	1689 (29.0%)	452 (29.3%)	616 (28.1%)	602 (30.1%)	19 (21.1%)
2011–2022	4141 (71.0%)	1092 (70.7%)	1580 (71.9%)	1398 (69.9%)	71 (78.9%)
Deprivation					
NZDep1‐4	2868 (49.2%)	768 (49.7%)	1050 (47.8%)	1012 (50.6%)	38 (42.2%)
NZDep5‐7	1653 (28.4%)	423 (27.4%)	637 (29.0%)	575 (28.8%)	18 (20.0%)
NZDep8‐10	1309 (22.5%)	353 (22.9%)	509 (23.2%)	413 (20.7%)	34 (37.8%)
Rurality					
Rural	796 (13.7%)	217 (14.1%)	309 (14.1%)	260 (13.0%)	10 (11.1%)
Urban/other	5034 (86.3%)	1327 (85.9%)	1887 (85.9%)	1740 (87.0%)	80 (88.9%)
Detection method					
Program screen	3522 (60.4%)	932 (60.4%)	1504 (68.5%)	1048 (52.4%)	38 (42.2%)
Non‐program image	1227 (21.0%)	341 (22.1%)	460 (20.9%)	413 (20.7%)	13 (14.4%)
Symptomatic	1081 (18.5%)	271 (17.6%)	232 (10.6%)	539 (27.0%)	39 (43.3%)
Laterality					
Left	3075 (52.7%)	849 (55.0%)	1131 (51.5%)	1049 (52.5%)	46 (51.1%)
Right	2755 (47.3%)	695 (45.0%)	1065 (48.5%)	951 (47.6%)	44 (48.9%)
DCIS grade					
High	2748 (47.1%)	297 (19.2%)	1190 (54.2%)	1234 (61.7%)	27 (30.0%)
Intermediate	2106 (36.1%)	672 (43.5%)	830 (37.8%)	579 (29.0%)	25 (27.8%)
Low	899 (15.4%)	544 (35.2%)	168 (7.7%)	173 (8.7%)	14 (15.6%)
Unknown	77 (1.3%)	31 (2.0%)	8 (0.4%)	14 (0.7%)	24 (26.7%)
DCIS size					
≤ 20 mm	3306 (57.6%)	1373 (88.9%)	1451 (66.1%)	482 (24.1%)	—
> 20 mm	2434 (42.4%)	171 (11.1%)	745 (33.9%)	1518 (75.9%)	—
Necrosis					
None	1883 (32.8%)	891 (57.7%)	552 (25.1%)	440 (22.0%)	—
Present	3492 (60.8%)	543 (35.2%)	1533 (69.8%)	1416 (70.8%)	—
Unknown	365 (6.4%)	110 (7.1%)	111 (5.1%)	144 (7.2%)	—
Facility of surgery					
Private	1741 (30.3%)	464 (30.1%)	663 (30.2%)	614 (30.7%)	—
Public	3735 (65.1%)	1001 (64.8%)	1458 (66.4%)	1276 (63.8%)	—
Unknown	264 (4.6%)	79 (5.1%)	75 (3.4%)	110 (5.5%)	
Surgical margin					
< 2 mm	750 (13.1%)	200 (13.0%)	356 (16.2%)	194 (9.7%)	—
≥ 2 mm	4923 (85.8%)	1318 (85.4%)	1827 (83.2%)	1778 (88.9%)	—
Clear, measurement unknown	67 (1.2%)	26 (1.7%)	13 (0.6%)	28 (1.4%)	—
ER status and adjuvant ET^a^					
ER‐negative	165 (2.8%)	15 (1.0%)	75 (3.4%)	73 (3.7%)	2 (2.2%)
ER‐positive with ET	195 (3.3%)	26 (1.7%)	109 (5.0%)	49 (2.5%)	11 (12.2%)
ER‐positive without ET	544 (9.3%)	147 (9.5%)	240 (10.9%)	153 (7.7%)	4 (4.4%)
Not tested	4926 (84.5%)	1356 (87.8%)	1772 (80.7%)	1725 (86.3%)	73 (81.1%)

Abbreviations: BCS: breast conserving surgery; ER: estrogen receptor; ET: endocrine therapy; RT: radiotherapy; SLNB: sentinel lymph node biopsy.

^a^ Endocrine therapy was recorded as adjuvant therapy in patients who underwent surgery and as primary therapy in those who did not.

### 3.2 Risk of Breast Cancer Events

**FIGURE 1:**
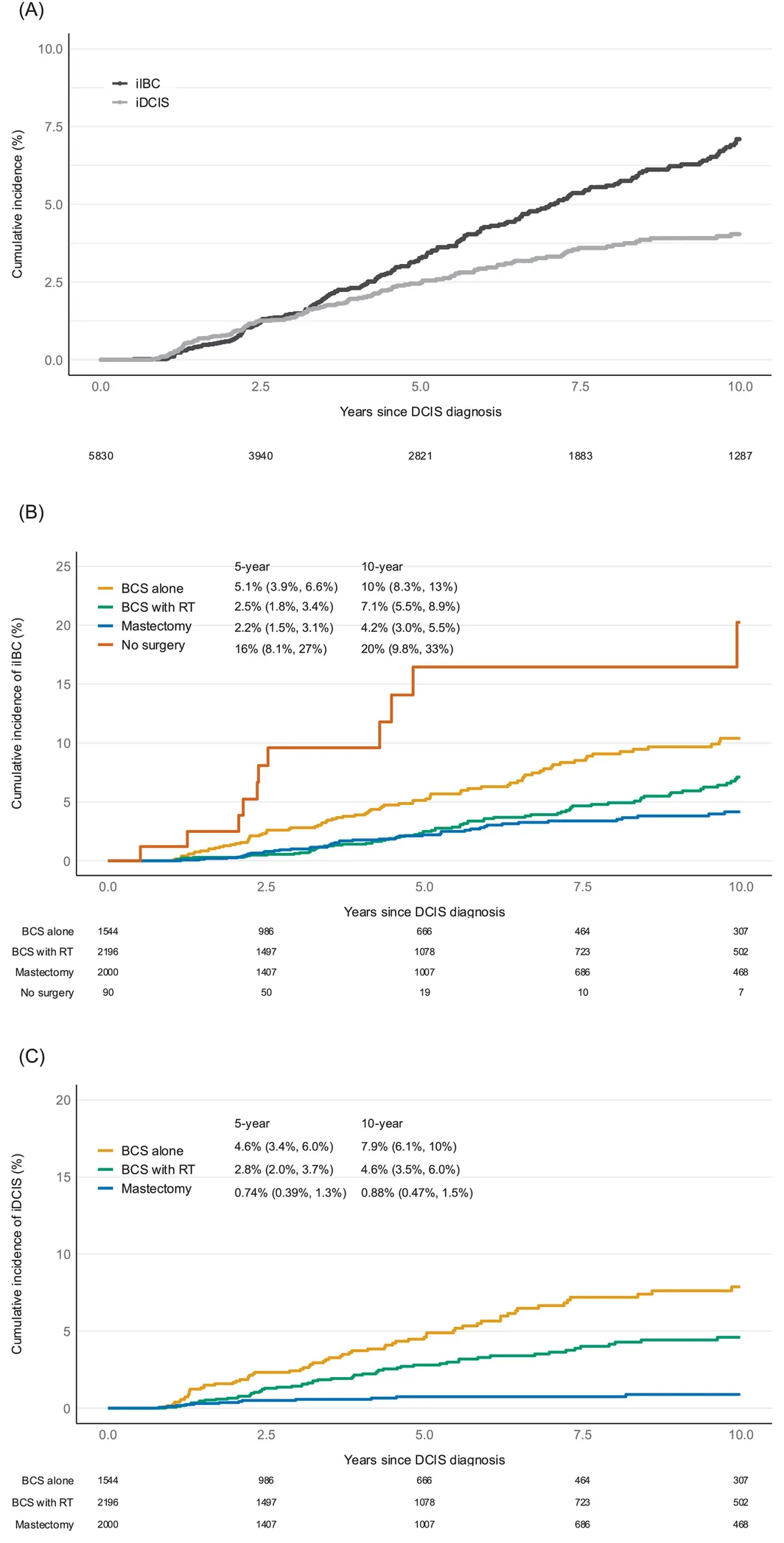
Cumulative incidence curves for ipsilateral invasive breast cancer and DCIS‐ipsilateral breast events in the overall cohort (A), for ipsilateral invasive breast cancer, by locoregional treatment (B), and for ipsilateral DCIS, by locoregional treatment (C).

During a median follow‐up of 4.8 (interquartile range, 1.7–9.3) years, 411 women (7.0%) developed IBE. The 5‐ and 10‐year cumulative incidences of iIBC in the overall cohort were 3.3% (95% CI: 2.7%, 3.9%), and 7.1% (95% CI: 6.2%, 8.1%), respectively (Figure 1A). The corresponding rates for iDCIS were 2.5% (95% CI: 2.0%, 3.0%), and 4.0% (95% CI: 3.4%, 4.8%). Cumulative incidences of iIBC and iDCIS differed by locoregional treatment group and were lowest in women receiving mastectomy or BCS with RT (Figure 1B,C). Following DCIS diagnosis, the cumulative incidence of iIBC continued to increase over time, while the incidence of iDCIS plateaued after 5 years in the overall cohort and across locoregional treatment groups. By diagnosis period, the 5‐year cumulative incidence of iIBC was lower in patients who were diagnosed in 2000–2010 compared to those diagnosed in 2011–2022 (Table S1). Similar patterns were observed in the two regions with data available from 2000 onward.

The 5‐ and 10‐year cumulative incidences of cIBC in the overall cohort were 2.6% (95% CI: 2.1%, 3.1%), and 5.9% (95% CI: 5.0%, 6.9%), respectively (Figure S2). The corresponding rates for cDCIS were 1.5% (95% CI: 1.2%, 1.9%) and 2.4% (95% CI: 1.8%, 3.0%). In terms of invasive breast cancer, including both iIBC and cIBC, the 5‐ and 10‐year cumulative incidences were 5.7% (95% CI: 5.0%, 6.5%) and 12.5% (95% CI: 11.3%, 13.8%), respectively.

**FIGURE 2:**
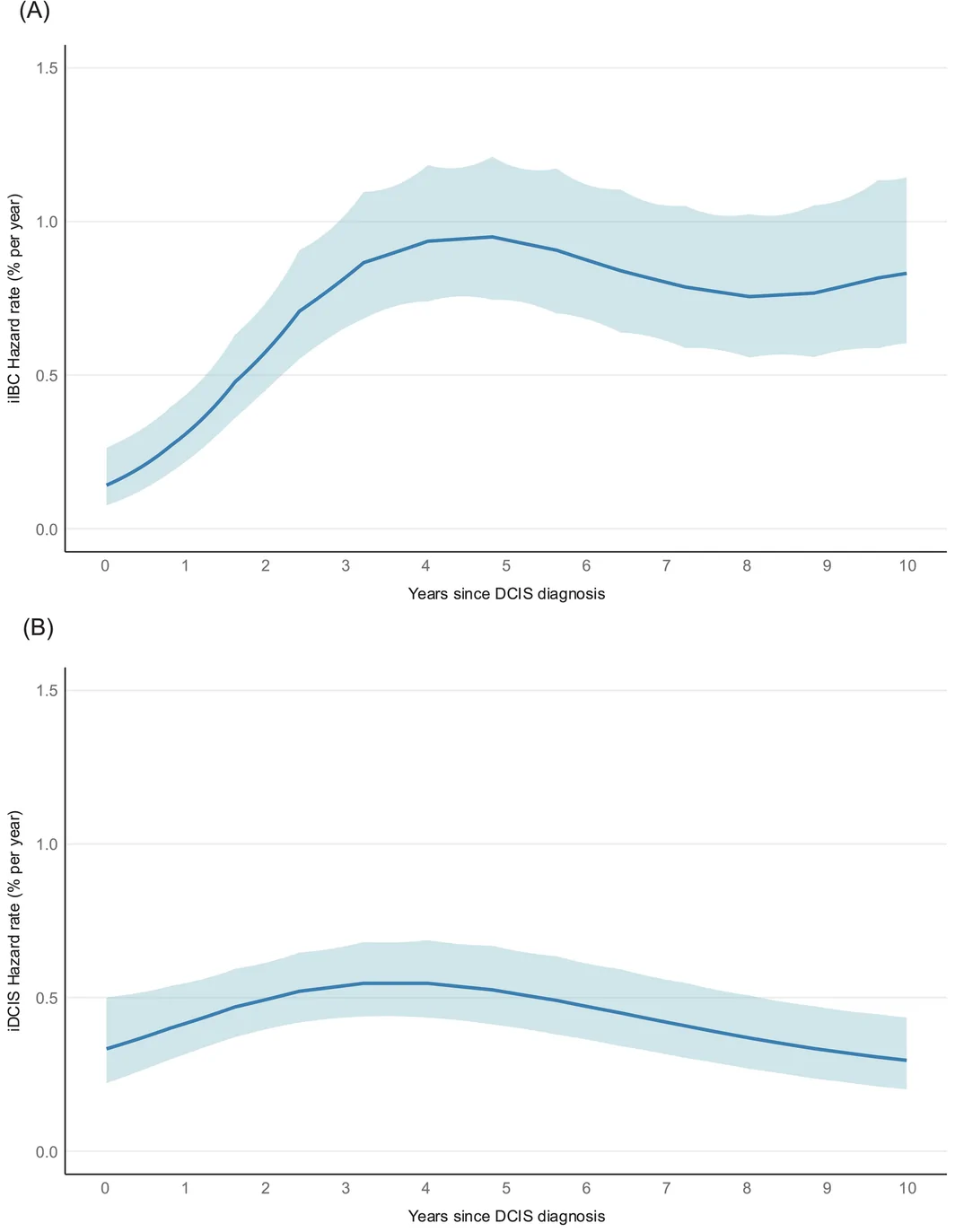
Hazard rate curves of ipsilateral invasive breast cancer (A) and ipsilateral DCIS (B) in the overall cohort.

In the overall cohort, the annual hazard rate of iIBC increased gradually over the first 5 years, reaching approximately 0.8% (Figure 2A). In contrast, the iDCIS hazard rates remained around 0.5% per year during the first 5 years and declined thereafter (Figure 2B). The hazard rates for iIBC differed by locoregional treatment group, with the highest rates observed among women who did not receive surgery, followed by those who received BCS alone. Rates were similar in the mastectomy and BCS with RT groups during the first 5 years, but increased thereafter in the BCS with RT group (Figure S3A). The hazard rates for iDCIS showed a similar pattern, except that the rate for women who received additional RT remained stable over time (Figure S3B).

The annual hazard rates of cIBC gradually increased to 0.7% at 10 years, while cDCIS remained stable at about 0.3% over time (Figures S4A and S4B).

### 3.3 Landmark Analysis

Among women who remained event‐free at 1, 3, and 5 years after DCIS diagnosis, the subsequent 5‐year cumulative incidence of iIBC and cIBC was stable over time. In contrast, the 5‐year incidence of iDCIS and cDCIS decreased (Table 2).

**TABLE 2:** Subsequent 5‐year cumulative incidence of breast events among women event‐free at each landmark year.

Landmark (years after diagnosis)	Number of patients at risk	Median follow‐up (years)	iIBC % (95% CI)	iDCIS % (95% CI)	cIBC % (95% CI)	cDCIS % (95% CI)
1‐year	4816	4.7	4.3 (3.6, 5.0)	2.8 (2.3, 3.4)	2.9 (2.4, 3.5)	1.4 (1.1, 1.8)
3‐year	3790	4.5	4.3 (3.5, 5.1)	2.4 (1.8, 3.0)	2.6 (2.1, 3.3)	1.0 (0.7, 1.5)
5‐year	2770	4.6	4.1 (3.3, 5.0)	1.7 (1.2, 2.3)	3.5 (2.8, 4.4)	0.9 (0.5, 1.4)

*Note:* median follow‐up was calculated from the landmark time.

Abbreviations: cDCIS, contralateral ductal carcinoma in situ; cIBC, contralateral invasive breast cancer; iDCIS, ipsilateral ductal carcinoma in situ; iIBC, ipsilateral invasive breast cancer.

Among women who received locoregional treatment (*n* = 5740), compared to women aged 45–69 at diagnosis, those aged < 45 years had a higher risk of iIBC in the overall cohort, which was slightly attenuated at the 5‐year landmark, whereas those aged > 69 years had a lower risk at the 5‐year landmark (Table 3). Women with DCIS size > 20 mm had a higher risk of iIBC in the overall cohort and at the 5‐year landmark. Compared to BCS alone, RT after BCS was associated with a lower risk in the overall cohort, but not at the 5‐year landmark. Mastectomy was associated with a consistently lower risk (Table 3).

**TABLE 3:** Multivariable Fine–Gray analysis of period of diagnosis and risk of ipsilateral invasive breast cancer in the overall cohort and 5‐year landmark cohort.

Variables	Overall cohort		5‐year landmark cohort	
	Events	sHR (95% CI)	Events	sHR (95% CI)
Age group				
< 45	32	**1.71 (1.12, 2.62)**	15	1.47 (0.80, 2.72)
45–69	198	Reference	119	Reference
> 69	22	0.90 (0.56, 1.45)	< 5	**0.23 (0.07, 0.77)**
Ethnicity				
European	192	Reference	106	Reference
Māori	22	1.17 (0.74, 1.86)	10	1.02 (0.52, 2.00)
Pacific	13	1.10 (0.60, 2.03)	9	1.56 (0.70, 3.47)
Asian	24	0.72 (0.47, 1.10)	11	0.64 (0.34, 1.19)
Deprivation				
NZDep1‐4	119	Reference	67	Reference
NZDep5‐7	74	1.11 (0.82, 1.51)	37	1.03 (0.68, 1.56)
NZDep8‐10	59	1.10 (0.77, 1.57)	33	1.18 (0.72, 1.94)
Rurality				
Urban/other	225	Reference	119	Reference
Rural	27	0.83 (0.56, 1.24)	18	1.01 (0.62, 1.66)
Detection method				
Program screen	114	Reference	61	Reference
Non‐program image	82	1.00 (0.72, 1.39)	51	0.88 (0.57, 1.34)
Symptomatic	56	1.20 (0.82, 1.77)	25	1.13 (0.65, 1.96)
DCIS size				
≤ 20 mm	157	Reference	89	Reference
> 20 mm	95	**1.52 (1.13, 2.04)**	48	**1.61 (1.08, 2.41)**
Grade				
High	99	Reference	45	Reference
Intermediate	101	1.06 (0.79, 1.43)	59	1.47 (0.97, 2.24)
Low	50	0.84 (0.54, 1.31)	33	1.62 (0.90, 2.92)
Necrosis				
Present	138	Reference	75	Reference
None	92	1.05 (0.75, 1.48)	48	0.79 (0.50, 1.25)
Unknown	22	0.77 (0.48, 1.24)	14	0.76 (0.42, 1.40)
Locoregional treatment				
BCS alone	98	Reference	47	Reference
BCS with RT	101	**0.63 (0.46, 0.87)**	67	0.98 (0.63, 1.53)
Mastectomy	53	**0.27 (0.18, 0.40)**	23	**0.28 (0.15, 0.51)**
Facility of Surgery				
Public	149	Reference	75	Reference
Private	90	1.20 (0.90, 1.60)	54	1.26 (0.85, 1.86)
Unknown	13	0.98 (0.53, 1.81)	8	1.74 (0.77, 3.90)
Surgical margin				
≥ 2 mm	206	Reference	110	Reference
< 2 mm	40	1.31 (0.93, 1.86)	22	1.45 (0.91, 2.31)
Clear, unknown measurement	6	1.20 (0.50, 2.90)	5	1.74 (0.65, 4.67)
ER status				
ER‐positive	31	Reference	14	Reference
ER‐negative	8	1.11 (0.50, 2.45)	4	1.19 (0.37, 3.84)
Not tested	213	1.07 (0.73, 1.57)	119	1.31 (0.74, 2.31)

*Note:* The sHRs were estimated using all follow‐up time for each cohort. Bold values denote statistical significance at the *p* < 0.05 level. The “Other/Unknown” ethnicity and “Unknown” DCIS grade categories were included for adjustment but are not shown due to small numbers.

Abbreviations: BCS: breast conserving surgery; ER: estrogen receptor; NZDep: New Zealand deprivation index; RT: radiotherapy; sHR: subdistributional hazard ratio.

### 3.4 Standardized Incidence Ratios of Invasive Breast Cancer

The rate of invasive breast cancer after a DCIS diagnosis was 14.0 per 1000 women per year in the overall cohort, and 13.8 per 1000 women per year among women diagnosed at screening age. The risk of invasive breast cancer was 5‐fold higher among women with DCIS compared to the general female population in New Zealand (SIR: 5.25; 95% CI: 4.79, 5.73). Similar results were observed when the analysis was restricted to women of screening age (SIR: 4.91; 95% CI: 4.45, 5.41).

### 3.5 Breast Cancer‐Specific Mortality and OS

By 31 December 2024, 531 patients had died, including 49 from breast cancer. The 5‐ and 10‐year cumulative breast cancer‐specific mortality rates were 0.2% (95% CI: 0.1%, 0.3%) and 0.7% (95% CI: 0.5%, 1.0%), respectively (Figure S5A). The 10‐year cumulative breast cancer‐specific mortality was highest among patients who did not receive surgery (8.8%; 95% CI: 3.5%, 17%), whereas rates were similar across BCS alone (0.31%; 95% CI: 0.09%, 0.87%), BCS with RT (0.38%; 95% CI: 0.15%, 0.87%), and mastectomy (1.1%; 95% CI: 0.63%, 1.7%) (Figure S5B). The 10‐year OS was 93.1% (95% CI: 92.3%, 93.9%) overall, with slightly higher OS in the BCS with RT group and lowest in non‐surgical patients (Figures S6A and S6B).

## 4 Discussion

In this cohort study of women in New Zealand with DCIS, we found that the risk of iIBC persisted beyond 5 years after DCIS diagnosis, whereas the risk of iDCIS declined over time. The higher iIBC risk associated with being aged under 45 at diagnosis attenuated at the 5‐year landmark, while the risk associated with DCIS ≥ 20 mm persisted. The lower iIBC risk associated with RT after BCS was not evident at the 5‐year landmark. Women with DCIS had elevated invasive breast cancer risk compared to the general population, but their overall risk of breast cancer‐specific mortality remained low.

We observed that the 5‐ and 10‐year cumulative incidences of invasive breast cancer after DCIS diagnosis were 5.7% and 12.5%, respectively. UK population‐based studies reported similar 5‐year rates (3%–7%), and slightly lower 10‐year rates (7.5%–12.0%), with rates varying by screening status [6, 7]. A pooled analysis of studies from Europe and the US reported a lower 10‐year iIBC rate of 3.2%, which may be because only 15% of patients were managed with BCS only. Although international studies with overlapping (1989–2021) and earlier (1978–2010) diagnosis periods have reported a decline in iIBC risk over time [26, 27], this pattern was not observed in our cohort. We found a lower 5‐year iIBC rate in the earlier period (2000–2010) overall and in the two regions with data available from 2000 onwards, which may partly reflect improved follow‐up procedures and more complete registry case capture over time. The 10‐year cumulative incidence of cIBC in our cohort was slightly higher than the estimates reported in the population‐based studies from the US [28], the UK [6, 7] and the Netherlands [29] (approximately 4%–5%).

The hazard function is useful for examining temporal variation in outcomes. Our study found that the hazard rates of iIBC increased in the first 5 years before stabilizing. This aligns with previous trials [11, 12] and cohort studies from the US [13] and the Netherlands [14], which compared BCS alone to BCS with RT and demonstrated that early reductions in iIBC risk were due to the addition of RT; without RT, the hazard rates of iIBC were consistent over time [11, 13]. This early protective effect of RT was also seen in a previous meta‐analysis of invasive breast cancer [30]. It is worth noting that in the studies with longer follow‐up (> 15 years) [12, 14], the hazard rates of iIBC following BCS alone and BCS with RT were similar at 10 years. Additionally, we observed that the hazard rates for iIBC were consistently low among patients who received mastectomy but were elevated in those who did not have surgery. These findings indicate that the benefit of RT is greatest in the first 5 years. Moreover, this effect has been shown to be modified by age, with a stronger effect observed among women diagnosed at age 50 years or older [10, 12].

In contrast, the hazard rates of iDCIS slightly decreased over time, with patients receiving RT after BCS consistently showing lower hazard rates than those treated with BCS alone. The decrease over time could be attributed to fewer patients remaining within the age range eligible for program screening after completing their 5‐year post‐surgery surveillance outside the program, thereby reducing the likelihood of detecting DCIS through mammography. Additionally, some residual DCIS may progress to iIBC over time, which aligns with evidence showing that approximately 80% of subsequent iIBCs are clonally related to the original DCIS [31]. Furthermore, the SweDCIS trial found that the protective effect of RT on iDCIS did not vary by age at diagnosis [12].

Consistent with the findings from a US study, the hazard rates of cIBC after DCIS increased over time, with a rate comparable to that observed in patients with invasive breast cancer [28]. We also observed that the hazard rates of cDCIS decreased over time.

The landmark breast event rates provide relevant prognostic information for patients who were event‐free for some time, which may support a more personalized follow‐up strategy. Results from the 1‐, 3‐, and 5‐year landmark analyses aligned with the hazard rate analyses, showing stable cumulative incidences of iIBC and cIBC and decreasing iDCIS and cDCIS over time. At the 5‐year landmark, the lower iIBC risk associated with adjuvant RT appeared attenuated, aligned with previous trials [12, 32]. The slightly attenuated and statistically non‐significant risks observed among younger women in our study might be due to a smaller sample size at the 5‐year landmark. A persistently higher risk was also observed for DCIS size ≥ 20 mm. Consistent with prior studies, no association between iIBC and DCIS grade was found in our cohort [27, 33].

Compared to the general population, women with a prior diagnosis of DCIS had a 5‐fold higher risk of developing invasive breast cancer. This elevated risk was similar to that observed in an Australian study (4‐fold higher) [34], but slightly higher than that reported in a United Kingdom study (2‐ to 4‐fold higher) [6, 7].

A recent US population‐based study reported that 5‐year iIBC was 8.1% among patients who did not receive upfront surgery within 12 months of diagnosis [35]. We found a slightly higher rate of 11.4% at 5 years in this study; however, the number of patients who received no surgery was very small.

Overall breast cancer mortality and OS in our cohort were comparable to other developed countries [5, 6, 7, 36, 37, 38]. Among patients who underwent surgery, no significant differences in breast cancer mortality were observed across different surgical groups, consistent with findings from a US study [5]. In line with findings from the US and South Korea, the improved OS was observed in patients who received BCS with RT compared to BCS alone, likely reflecting both the reduction in local recurrence with RT and treatment selection bias favoring healthier patients [39, 40]. The breast cancer mortality rate observed in our non‐surgical patients was similar to those previously reported in the United States (6.6%) [37]. However, survival estimates for the no surgery group in our study should be interpreted cautiously given the small sample size.

In New Zealand [2], similar to the UK [41] and the Nertherlands [42], annual mammogram surveillance is recommended for the initial 5 years after diagnosis of DCIS, followed by routine program screening. In practice, physicians may recommend ongoing annual mammogram surveillance for women who remain in good health. Our findings suggest that the risk of subsequent iIBC varies over time, and the similar 10‐year cumulative risk observed for cIBC and iIBC collectively supports the potential benefit of continued mammogram surveillance, particularly bilateral mammography when both breasts are present. Although treatment with BCS alone or BCS with RT did not affect survival, women remained at an elevated long‐term risk beyond 5 years after DCIS diagnosis.

To our knowledge, this is the first study to examine the temporal changes in subsequent breast cancer outcomes among women with DCIS in New Zealand. Despite covering only 63% of national cases before 2020, our cohort remains nationally representative as it includes the 4 major treatment centers. The registry's ability to integrate data from the national cancer registry and several other sources enhances the comprehensiveness of the cohort. Several limitations should be noted. DCIS was defined using a diagnosis code, which may have inadvertently included mixed in situ lesions. Because the data collection commenced at different times across regions, some subsequent events may not have been captured for patients who migrated between regions during or after treatment [43]. This has also limited our ability to examine temporal differences in outcomes by diagnosis period. As ER testing and the use of ET remained low, the impact of ET on the temporal pattern of subsequent events could not be evaluated. Additionally, data on lifestyle factors, such as smoking, alcohol intake and body mass index, were not available for the majority of patients. Future studies with longer follow‐up are warranted given the smaller sample size for the 5‐year landmark cohort.

In conclusion, the risk of iIBC after DCIS persisted among women who were event‐free at 5 years. Continued long‐term mammographic surveillance as part of the follow‐up strategy is important for women with larger DCIS, those treated with BCS with or without RT, and possibly younger women.

## Author Contributions

**Qian Chen:** conceptualization, methodology, data curation, formal analysis, writing – original draft, writing – review and editing. **Ian Campbell:** writing – review and editing. **Mark Elwood:** writing – review and editing. **Alana Cavadino:** writing – review and editing. **Phyu Sin Aye:** writing – review and editing. **Sandar Tin Tin:** writing – review and editing, conceptualization, project administration, funding acquisition.

## Funding

This study was supported by Auckland Medical Research Foundation (Ref: 1124002) and University of Auckland Research Development Fund (Ref: 3729227). The funding sources had no direct involvement in this study or the decision to submit the paper for publication.

## Ethics Statement

This study was approved by the Auckland Health Research Ethics Committee (Ref. AH26746).

## Conflicts of Interest

The authors declare no conflicts of interest.

## Supporting information


**Figure S1:** Flow chart of cases selection.
**Figure S2:** Cumulative risk curves of contralateral invasive breast cancer and contralateral DCIS for the overall cohort.
**Figure S3:** Hazard rate curves of ipsilateral invasive breast cancer (A) and ipsilateral DCIS (B), by locoregional treatment group.
**Figure S4:** Hazard rate curves of contralateral invasive breast cancer (A), contralateral DCIS (B) in the overall cohort.
**Figure S5:** Breast cancer‐specific mortality among women with DCIS in overall cohort (A), by locoregional treatment (B).
**Figure S6:** Overall survival among women with DCIS in overall cohort (A), by locoregional treatment (B).
**Table S1:** Five‐year cumulative incidence of ipsilateral invasive breast cancer by diagnosis period and region.

## Data Availability

All source code is publicly available on GitHub (https://github.com/DCIS‐NZ/DCIS_outcomes). The datasets used in this study may be requested from the Breast Cancer Foundation National Register New Zealand. Deidentified data and further information that support the findings of this study are available from the corresponding authors upon request.
