# Supplementary material for: Breast Cancer Outcomes From Ductal Carcinoma In Situ: A Population‐Based Cohort Study
Source: Int J Cancer. 2026 May 20;159(8):1917–27. doi: 10.1002/ijc.70559 (PMC13495889; doi:10.1002/ijc.70559)
Supplement: Supplementary file 1 [file IJC-159-1917-s001.pdf]

Supplementary Material

Breast cancer outcomes from ductal carcinoma in situ: a population-based cohort study

Qian Chen, Ian Campbell, Mark Elwood, Alana Cavadino, Phyu Sin Aye, Sandar Tin Tin

Table of contents

Supplementary figures

Figure S1 Flow chart of cases selection. 2
Figure S2 Cumulative risk curves of contralateral invasive breast cancer and contralateral DCIS for the overall cohort. 2
Figure S3 Hazard rate curves of ipsilateral invasive breast cancer (A) and ipsilateral DCIS (B), by locoregional treatment group. 3
Figure S4 Hazard rate curves of contralateral invasive breast cancer (A), contralateral DCIS (B) in the overall cohort. 4
Figure S5 Breast cancer-specific mortality among women with DCIS in overall cohort (A), by locoregional treatment (B). 5
Figure S6 Overall survival among women with DCIS in overall cohort (A), by locoregional treatment (B). 6

Supplementary tables

Table S1 Five-year cumulative incidence of ipsilateral invasive breast cancer by diagnosis period and region. 6

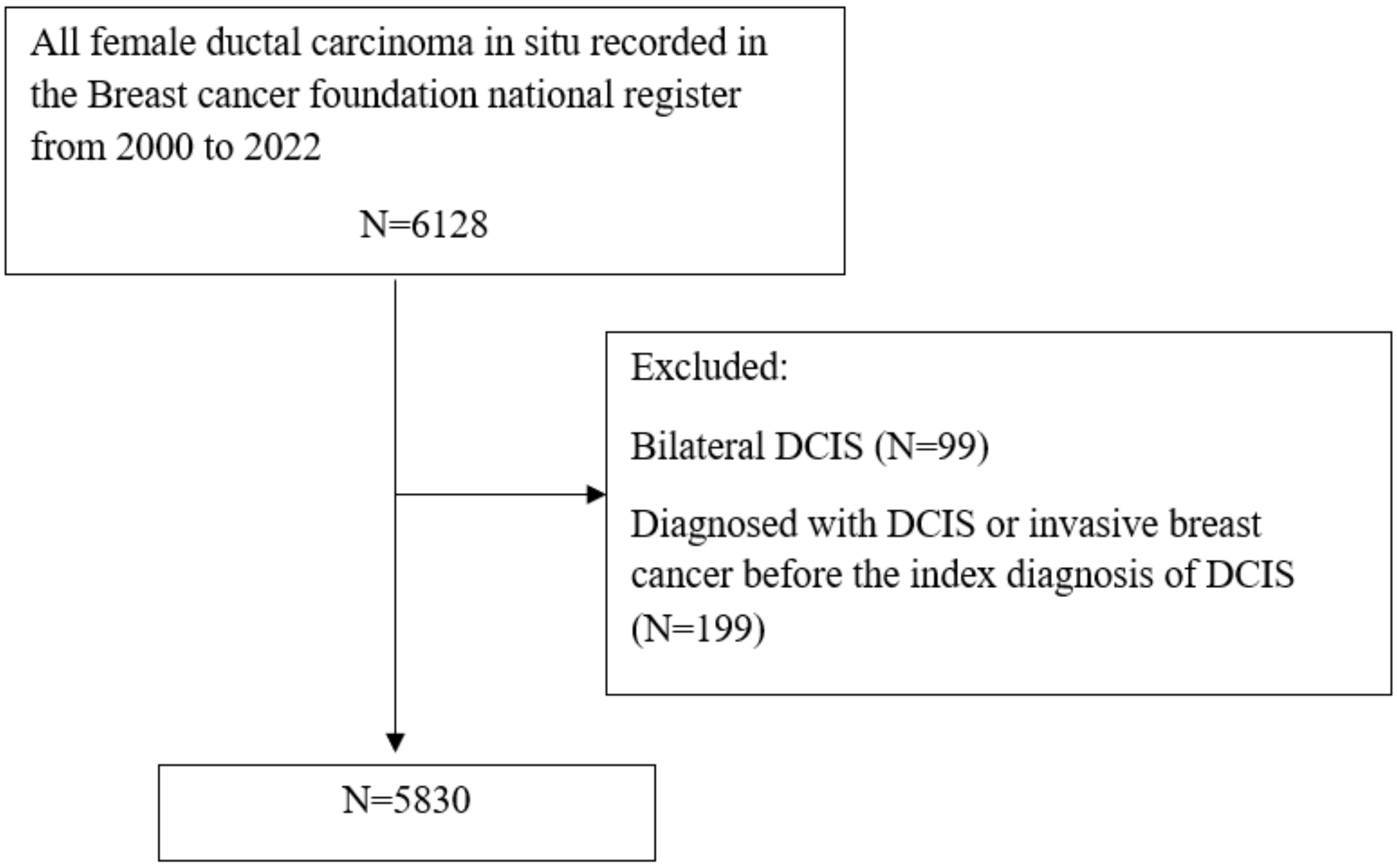

Figure S1 Flow chart of cases selection.

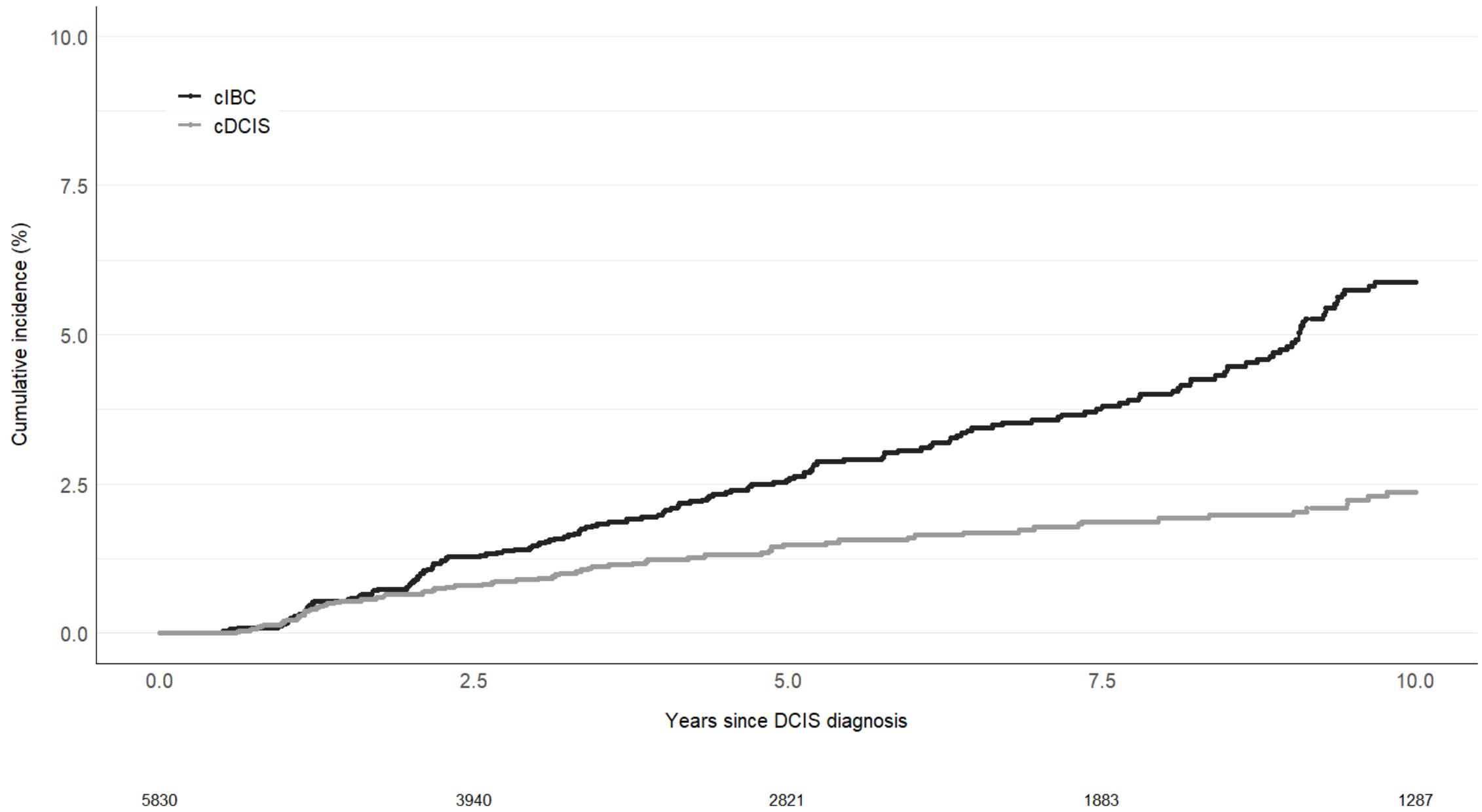

Figure S2 Cumulative risk curves of contralateral invasive breast cancer and contralateral DCIS for the overall cohort.

(A)

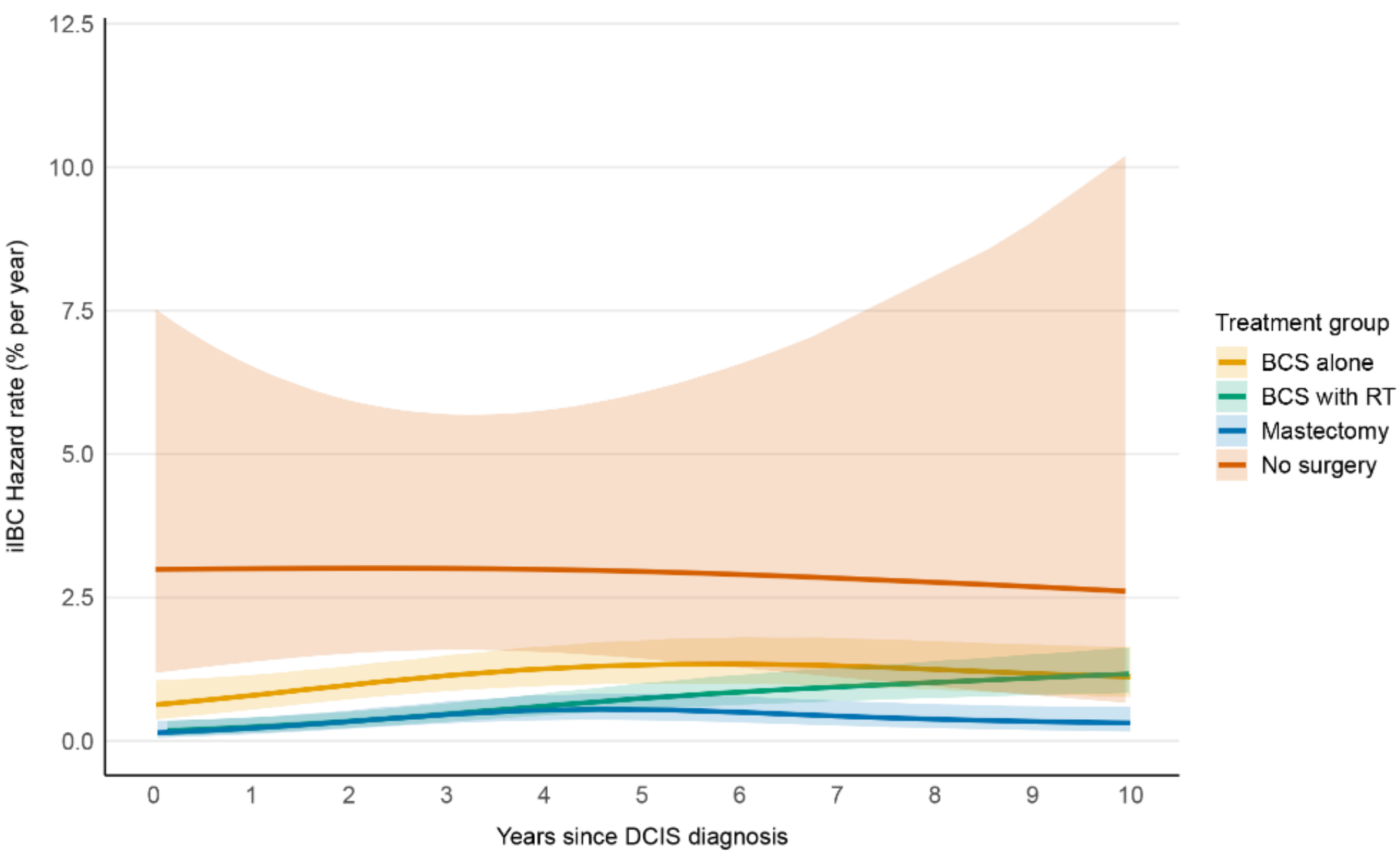

(B)

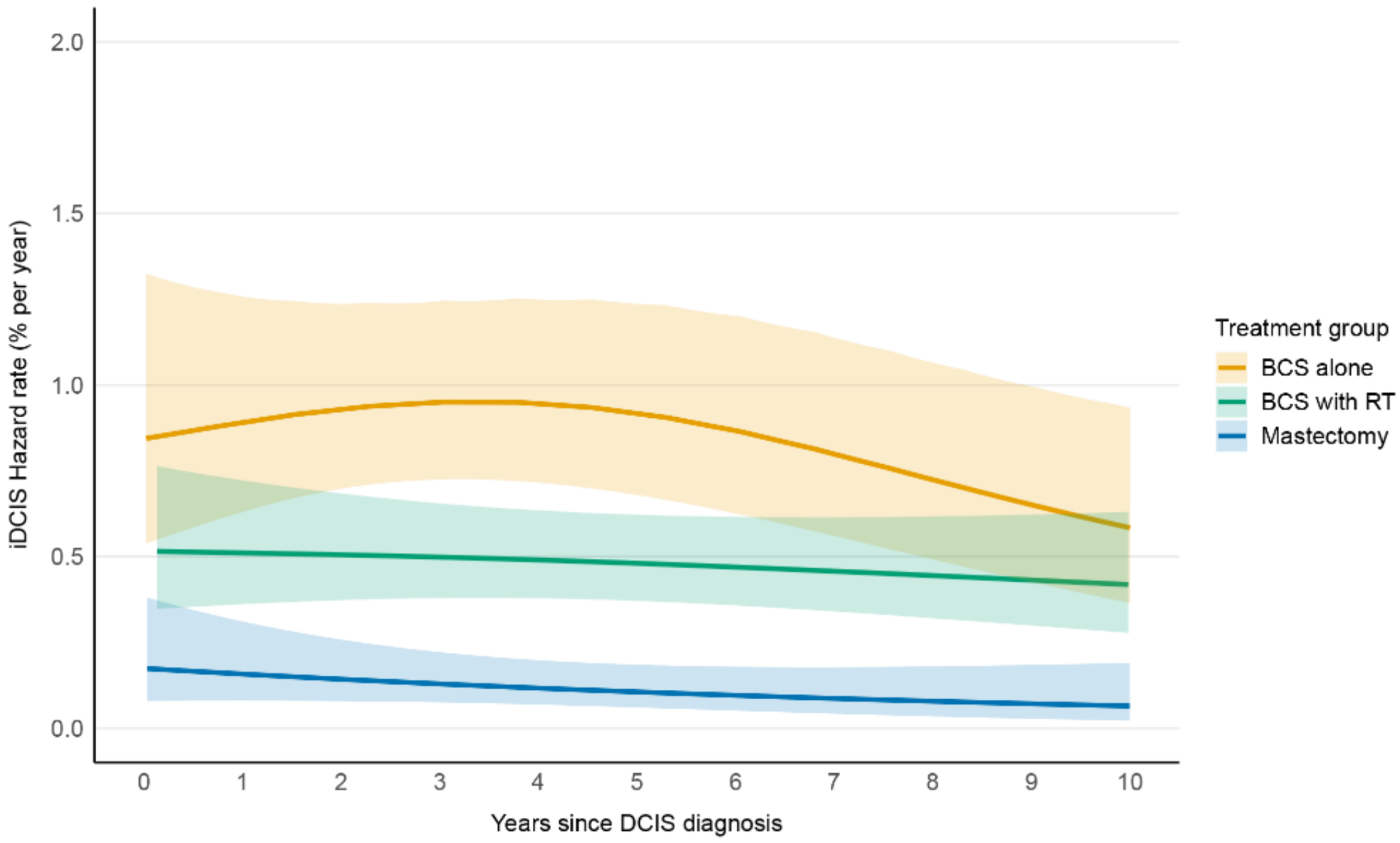

Figure S3 Hazard rate curves of ipsilateral invasive breast cancer (A) and ipsilateral DCIS (B), by locoregional treatment group.

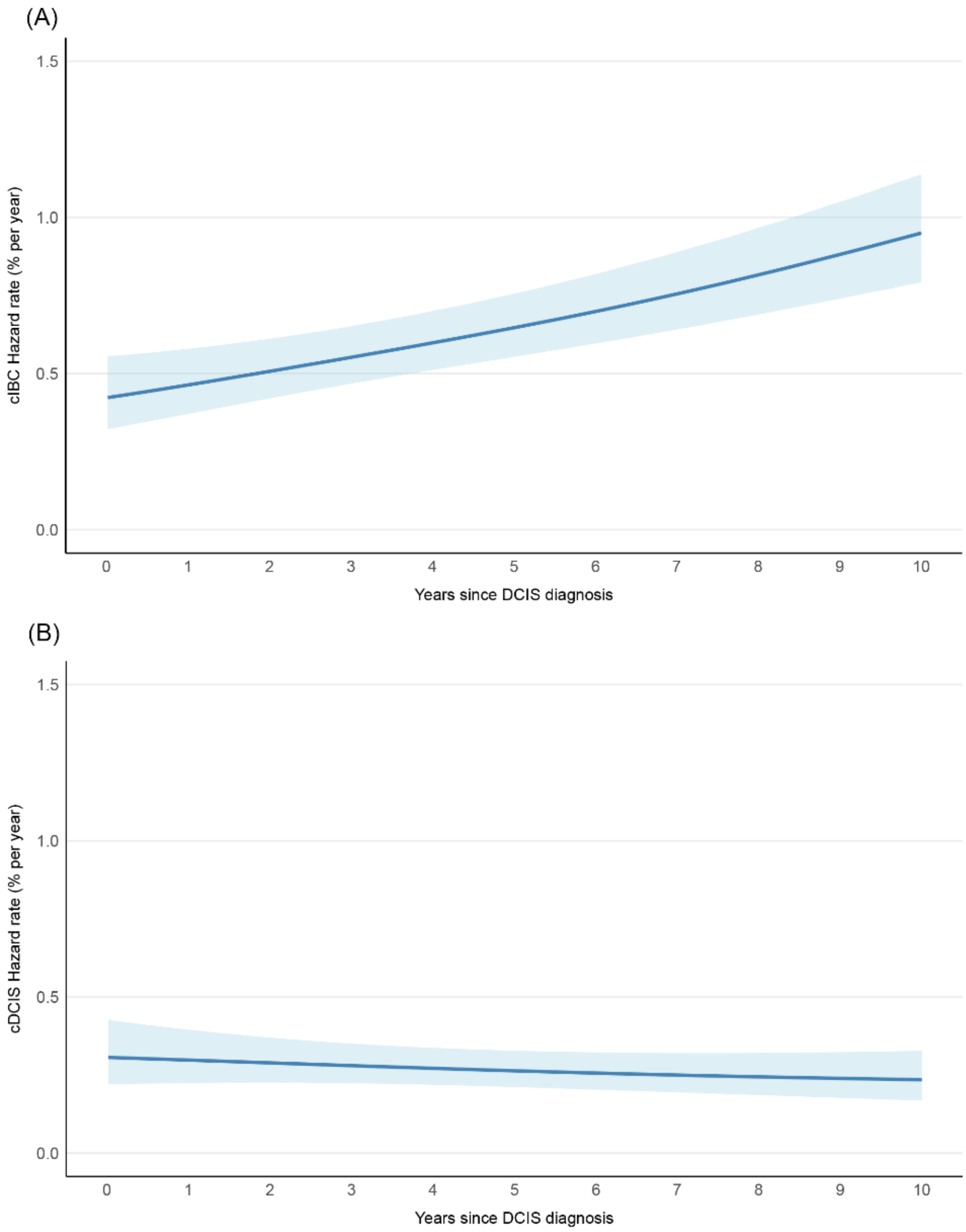

Figure S4 Hazard rate curves of contralateral invasive breast cancer (A), contralateral DCIS (B) in the overall cohort.

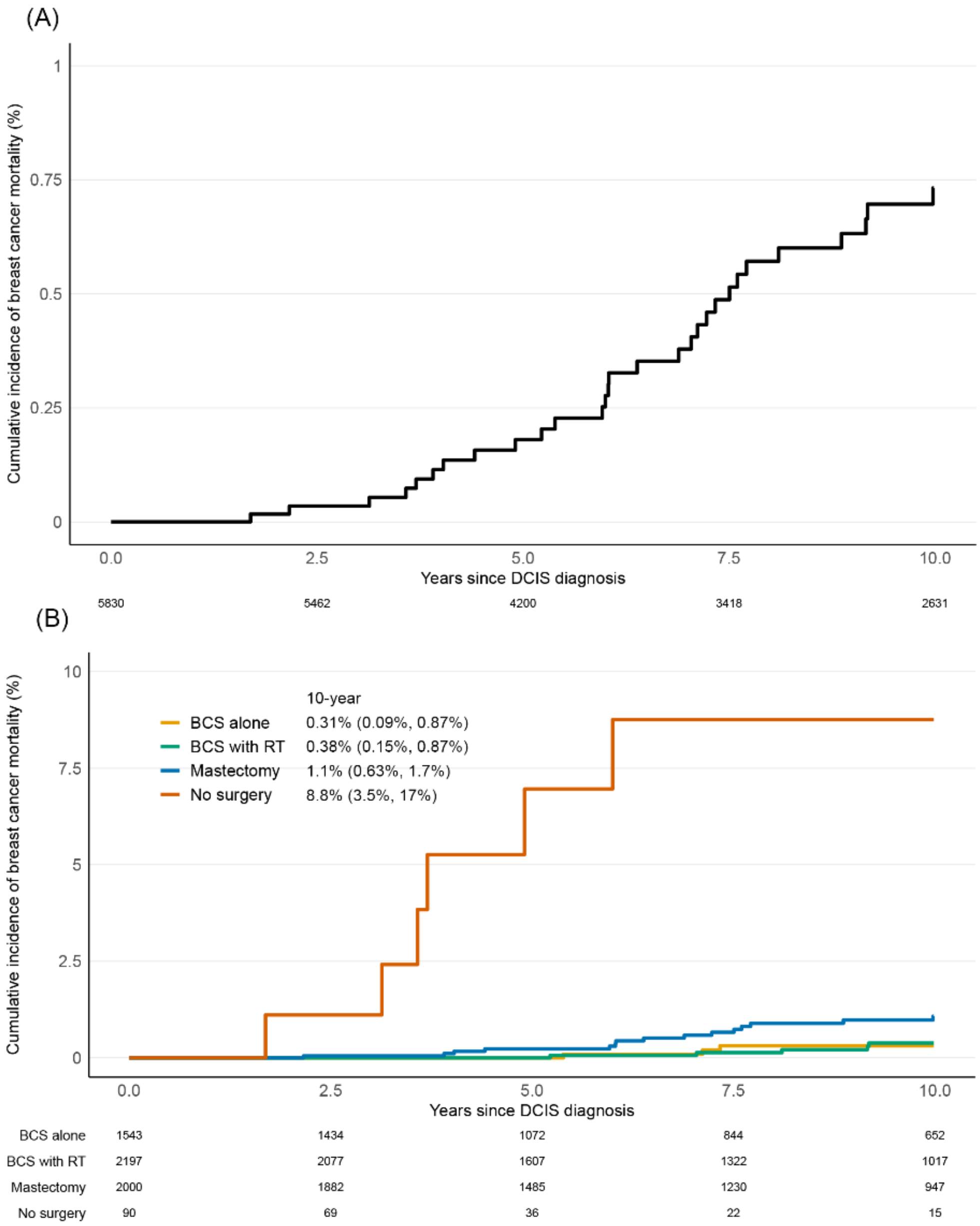

Figure S5 Breast cancer-specific mortality among women with DCIS in overall cohort (A), by locoregional treatment (B).

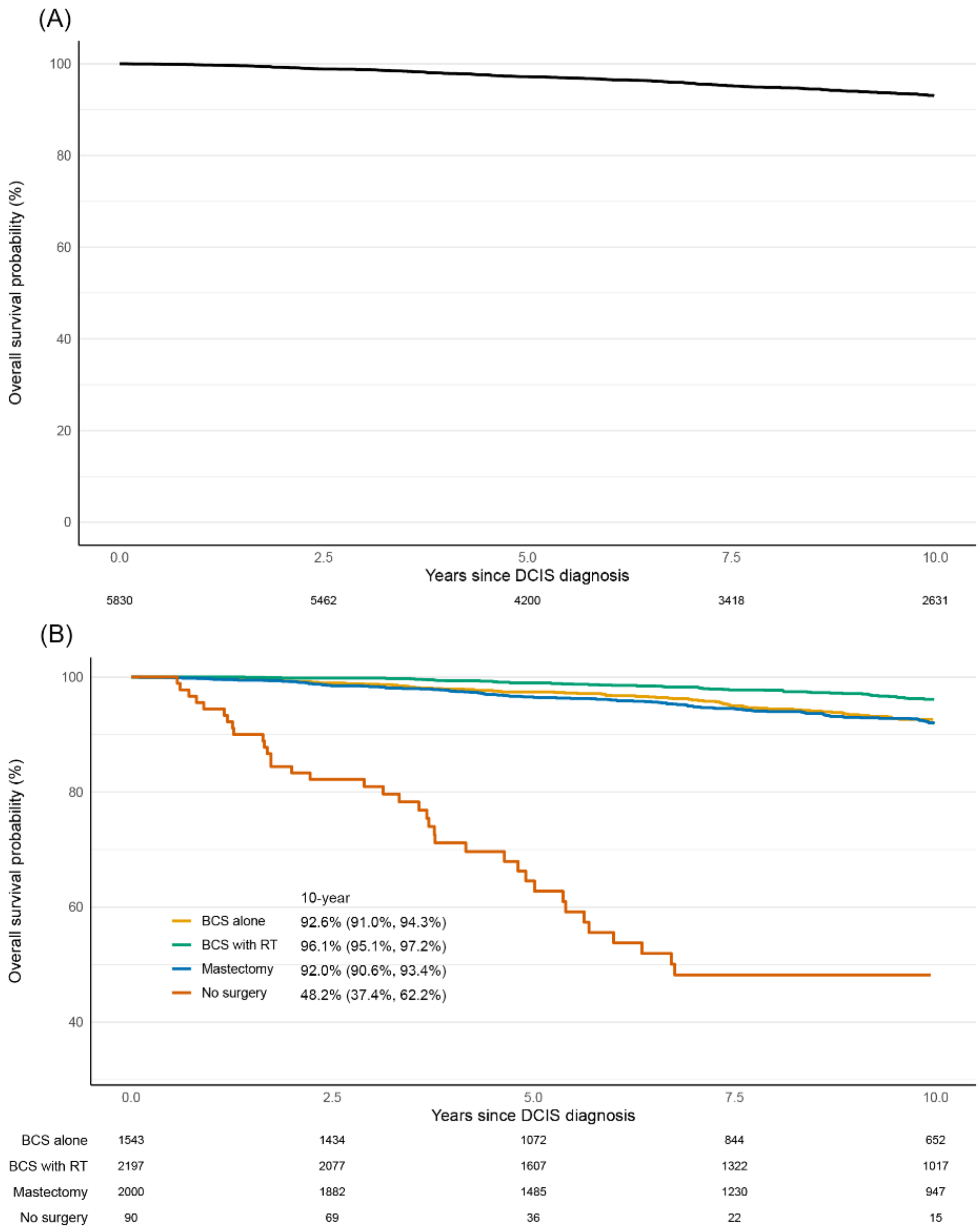

Figure S6 Overall survival among women with DCIS in overall cohort (A), by locoregional treatment (B).

Table S1 Five-year cumulative incidence of ipsilateral invasive breast cancer by diagnosis period and region.

Region	2000-2010	2011-2022
Overall<br>N=5830	2.6% (95%CI: 1.9%, 3.4%)	3.9% (95%CI: 3.1%, 4.7%)
Auckland and Waikato<br>N=3835	2.6% (95%CI: 1.8%, 3.4%)	3.5% (95%CI: 2.6%, 4.7%)
Auckland<br>N=3104	2.4% (95%CI: 1.7%, 3.4%)	3.4% (95%CI: 2.5%, 4.7%)
Waikato<br>N=731	3.1% (95%CI: 1.5%, 5.8%)	4.0% (95%CI: 2.0%, 7.0%)
